# The double-edged sword of statins in intracerebral hemorrhage patients: a systematic review and meta-analysis

**DOI:** 10.3389/fneur.2025.1519818

**Published:** 2025-01-27

**Authors:** Zheng Li, Wen-qi Xu, Jiao-qi Wang, Jia-hui Yang, Xiao-hua Shi, Cheng-bing Wang, Zhong-xin Xu, Jin-lan Jiang

**Affiliations:** ^1^Department of Neurology, China-Japan Union Hospital of Jilin University, Changchun, China; ^2^Scientific Research Center, China-Japan Union Hospital of Jilin University, Changchun, China

**Keywords:** statin, intracerebral hemorrhage, mortality, meta-analysis, functional prognosis

## Abstract

**Background:**

This meta-analysis aimed to investigate the effect of statins on the prognosis of patients with intracerebral hemorrhage (ICH).

**Methods:**

We conducted a systematic search using the keywords “statin” and “intracerebral hemorrhage” across four electronic databases (PubMed, Cochrane Library, Web of Science, and Embase) from their inception to October 31, 2023, to identify studies comparing the effects of statins on the prognosis of patients with ICH. The primary outcome was total mortality after ICH. This meta-analysis was registered online (PROSPERO ID: CRD42023493063).

**Results:**

Our initial search identified 5,543 studies. After applying inclusion criteria, 30 studies with a total of 42,298 patients were included in the final analysis. Our meta-analysis showed that statins significantly reduced overall mortality in patients with ICH (OR: 0.61; 95% CI: 0.51–0.73; *I*^2^ = 87%; *p* < 0.01). Subgroup analyses further demonstrated lower mortality in ICH patients treated with statins compared to those not treated, including in the propensity score matching (PSM) group (OR: 0.59; 95% CI: 0.48–0.74; *I*^2^ = 90%; *p* < 0.01), the prospective cohort study (PCS) group (OR: 0.56; 95% CI: 0.40–0.77; *I*^2^ = 89%, *p* < 0.01), and the retrospective cohort study (RCS) group (OR: 0.64; 95% CI: 0.51–0.81; *I*^2^ = 87%, *p* < 0.01).

**Conclusion:**

Our meta-analysis of 30 studies suggests that statin use may be associated with improved mortality and functional outcomes in patients with intracerebral hemorrhage (ICH).

**Systematic Review Registration:**

https://www.crd.york.ac.uk/PROSPERO/, CRD42023493063.

## Introduction

Intracerebral hemorrhage (ICH) is associated with high disability (42%) and mortality rates (50%), creating a substantial burden on families and society (1, 2). In clinical practice, patients prior to ICH with ischemic cardiovascular and cerebrovascular diseases frequently use statins as primary and secondary prevention for ischemic stroke (3). However, the lipid-lowering effect of statins could be a double-edged sword (1). While statins reduce the occurrence of endothelial atherosclerosis and possess multiple antioxidative properties, they may also compromise vascular endothelial integrity, potentially increasing the risk of hemorrhage.

The Stroke Prevention by Aggressive Reduction in Cholesterol Levels (SPARCL) trial (NCT00147602), a randomized controlled trial (RCT) involving 4,731 patients and published in 2006 (4), demonstrated that atorvastatin significantly reduced the incidence of stroke and transient ischemic attack (TIA). Conversely, a secondary analysis of SPARCL in 2009 revealed that statins might increase the risk of spontaneous intracerebral hemorrhage (5). This finding prompted many clinicians to reconsider the safety of statin use in patients with a history of ICH. Subsequent research from 2010 to 2015, including 13 observational cohort studies and five meta-analyses (6–10), generally concluded that statins do not worsen the prognosis of cerebral hemorrhage. However, these meta-analyses often lacked sufficient sample sizes and robust subgroup analyses to adequately control for confounding factors. Consequently, it remains uncertain whether patients with ICH and concurrent ischemic cardiovascular disease can safely use statins for ischemic stroke prevention.

Given these uncertainties, it is imperative to incorporate the latest research into a comprehensive meta-analysis to better guide clinicians in developing more rational statin treatment strategies for patients with hemorrhagic stroke.

## Methods

### Search strategy and selection criteria

This meta-analysis is reported in accordance with the Meta-Analysis (PRISMA) Statement and followed the PICO principle (P: Patients with spontaneous intracranial hemorrhage, I: statin treatment; C: non-statin treatment, and O: prognosis of spontaneous intracerebral hemorrhage). In order to improve the quality of our analysis, we formulated a series of strict inclusion and exclusion criteria to ensure that only the most relevant studies were included in our analyses.

Eligible studies were observational cohort studies or randomized controlled trial (RCT) studies that conformed to the following criteria: (1) case–control or cohort studies that investigated the effect of statins on the prognosis of patients with ICH by comparing a statin-treated group and a non-statin-treated group; (2) head computed tomography (CT) or magnetic resonance imaging (MRI) that clearly met the imaging diagnostic criteria for ICH; (3) no significant differences between the statin group and non-statin group in terms of gender, mean age, and medical history; (4) a body of research data that could be extracted; (5) clear outcome indicators; (6) the full text is available and supplementary information could be obtained from the authors to obtain additional materials and data if required, and (7) published in English.

Our exclusion criteria were as follows: (1) the research content was not related to the effect of statins on ICH; (2) a lack of head CT scans or MRI data as diagnostic criteria for ICH; (3) incomplete records of ICH prognosis; (4) patients with traumatic subarachnoid hemorrhage, intracranial aneurysm rupture bleeding, venous thrombolysis related bleeding, bleeding after mechanical thrombectomy, cerebral infarction bleeding transformation; (5) article was a summary, review, case report, lecture, reply to the editor, or Trail registry record; (6) animal experiments, and (7) articles for which the full text was not available, data are not available, no clear outcome measures, and articles for which the authors could not be contacted.

By applying Boolean logic operations, we searched four key online databases (PubMed, Embase, the Cochrane Library and the Web of Science) for ‘intracerebral hemorrhage’ and ‘statin’. This systematic literature search was conducted on the 31st of October 2023 (For detailed information on subject words, free words, and search terms, please refer to the Supplementary material). We restricted our search to articles published in English. Our meta-analysis protocol was registered online (PROSPERO ID: CRD42023493063) and followed a prespecified plan of analysis.

### Study selection and data extraction

Two investigators (JQ-W and JH-Y) independently screened the titles, abstracts and full texts of the articles identified by our literature search. Irrelevant and duplicate publications were rejected to ensure that the remaining articles met our inclusion criteria. Disagreements were resolved by consensus or by consulting a senior reviewer (XH-S). Next, we extracted specific data from the original research articles, as required. Data extraction was performed independently and in duplicate by two reviewers (CB-W and JL-J) using a predetermined data-extraction table which included the following information: including basic features (author, publication date, the country in which the subjects resided, the number of patients involved in the statin and non-statin groups, trial duration, mean population age, and male proportion), intervention characteristics (statin type, dose, time), study design (study center, propensity score matching (PSM), follow-up times, bias) and outcomes (outcome indicators, primary outcome).

### Risk of bias assessment

Two of the authors (ZL and WQ-X) independently reviewed the risk of bias in the included observational cohort studies by using the Newcastle-Ottawa Scale (NOS) and in the RCTs by using the updated version of the Cochrane Risk of Bias Tool. Articles with a NOS score > 7 were considered to represent high-quality research studies and were included in our meta-analysis. Disagreements between the reviewers were resolved by discussion and a third reviewer (XZ-X) was consulted to reach a consensus.

### Strategy for data synthesis

Binary classification variables were compared by odds ratios (ORs) and 95% confidence intervals (CIs). The standard *I*^2^ test was used to evaluate heterogeneity between the studies; an *I*^2^ > 50% indicated that a random effect model (RM) should be applied, while an *I*^2^ < 50% indicated that the fixed effect model (FM) should be applied. Following the generation of forest and funnel maps, research studies with higher levels of heterogeneity were eliminated and the data were re-analyzed. All calculations were performed using statistical software provided by the Cochrane Collaboration (RevMan 5.3) and R 4.3.2.

### Primary outcome and subgroup analyses

The primary outcome of our meta-analysis was total mortality after ICH, and the secondary outcome was functional prognosis. In order to improve the consistency of data between groups, we grouped patients with different study conditions for analysis. Our subgroup analysis included mortality at different times after the occurrence of ICH [period of hospitalization: 30 days, 90 days, and long term (0.5–1 year)], and a better functional prognosis (a modified Rankin Scale [mRS] score of 0–3) at different times after the occurrence of ICH (at discharge, 90 days, and long term). In addition, observational cohort studies were divided into several subgroups according to their characteristics: design (prospective cohort study, retrospective cohort study) and propensity score matching.

## Results

### Study selection and characteristics

**Figure 1:**
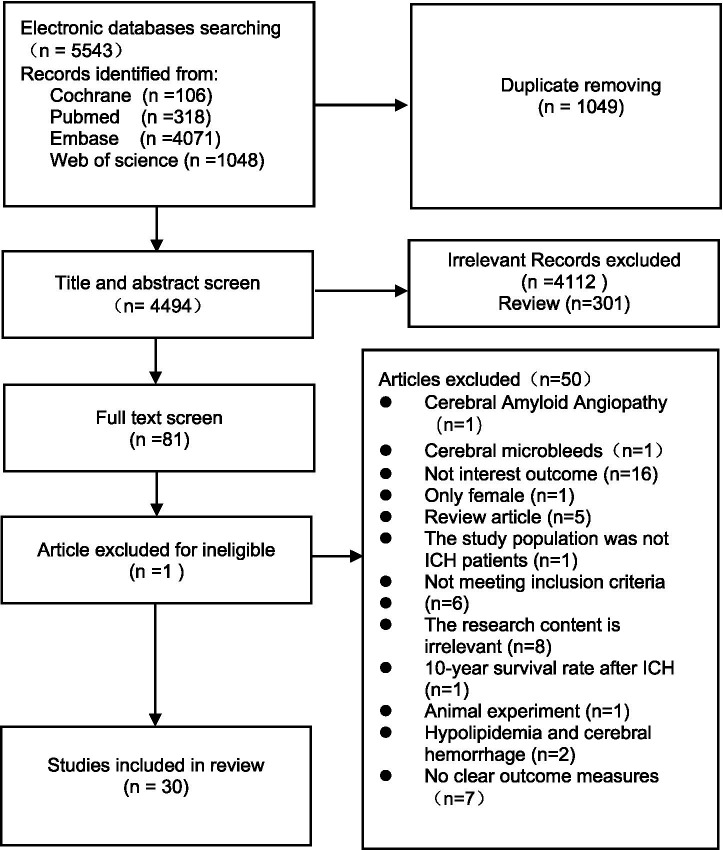
Flow diagram of literature searching and selection. ICH, intracerebral hemorrhage.

Initially, our literature search identified 5,543 reports; however, 1,049 of these articles were removed due to duplication. After screening the titles and abstracts, we removed a further 4,112 reports that were irrelevant to our research focus, and 301 reviews. After screening the full text of the remaining 81 articles, 50 articles were excluded as they were unavailable. Supplementary Table S1 lists the specific reasons for exclusion. It was not possible to extract key data from Wubshet et al. (11) due to the lack of detailed information relating to the number of deaths from ICH after taking statins. Finally, 30 eligible studies (5, 10, 12–39) were included in our final meta-analysis. Figure 1 depicts the process used to retrieve data. Our analysis included 42,298 people, including 13 articles (10, 14, 15, 22, 26–28, 31, 32, 36, 38, 39) from the Americas, five articles (12, 17, 23, 29, 34) from Europe, eight articles (13, 16, 19–21, 24, 25, 30) from Asia, two articles (35, 37) from the Middle East, and two international articles (5, 18). In addition, we included eight studies (13, 15, 16, 19–22, 24) that contained propensity score matching and detailed information relating to other key experimental characteristics, such as population. Design and outcome indicators are shown in Supplementary Table S2. With regards to the quality of the 30 research articles included in this meta-analysis, 28 observational cohort studies were analyzed by the NOS rating scale (7–9 points), and two RCT studies (5, 14) were analyzed by the Cochrane risk assessment tool. All of the studies included in our analysis were high-quality studies with a low risk of bias.

### Primary outcome: total mortality

**Figure 2:**
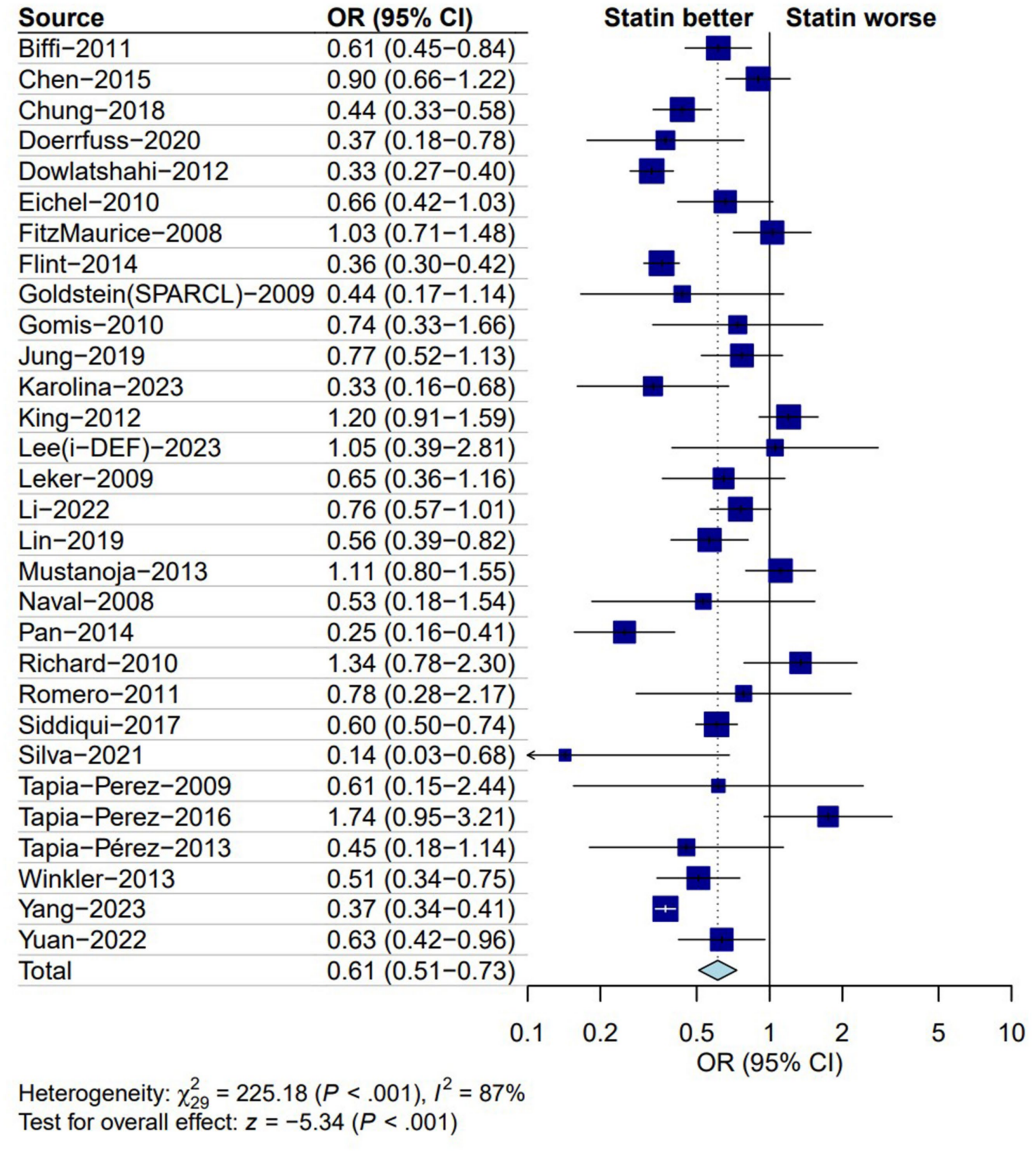
Forest plot of total mortality between statin and non-statin group. OR, odds ratio; CI, confidence interval.

All 30 of the studies included in our analysis included total mortality as the primary outcome measure. There was a significant reduction in the total mortality of patients with ICH who received statins compared to those who did not (OR: 0.61; 95% CI: 0.51–0.73; *I*^2^ = 87%; *p* < 0.01; Figure 2). Subgroup analysis at other independent time points revealed that compared with patients with ICH who did not receive statins, those who did receive statins during hospitalization showed a reduction in mortality (OR: 0.63, 95%CI: 0.46–0.88; *I*^2^ = 74%; *p* < 0.01; Supplementary Figure S3). When compared with patients with ICH who did not receive statins, those who did received statins 30 days after ICH showed a reduction in mortality (OR: 0.52; 95% CI: 0.29–0.92; *I*^2^ = 93%; *p* = 0.03; Supplementary Figure S4). When evaluated 90 days after ICH, patients who received statins showed a reduction in mortality when compared to those who did not (OR: 0.62; 95% CI: 0.47–0.82; *I*^2^ = 70%; *p* < 0.01; Supplementary Figure S5). The long-term administration of statins after ICH reduced the mortality of ICH patients when compared to non-administration (OR: 0.54; 95% CI: 0.33–0.91; *I*^2^ = 91%; *p* = 0.02; Supplementary Figure S6).

### Subgroup analysis: quality and design of the included studies

Our subgroup analysis also included experimental quality and design. Compared with patients with ICH who did not receive statins, the propensity score matching group showed a significant reduction in mortality (OR: 0.59; 95% CI: 0.48–0.74; *I*^2^ = 90%; *p* < 0.01; Supplementary Figure S7). Patients with ICH in the prospective cohort study group who received statins showed a significant reduction in mortality when compared with those who did not receive statins (OR: 0.56; 95% CI: 0.40–0.77; *I*^2^ = 89%; *p* < 0.01; Supplementary Figure S8). Furthermore, patients with ICH in the retrospective cohort study group who received statins showed a reduction in mortality when compared to those who did not receive statins (OR: 0.64; 95% CI: 0.51–0.81; *I*^2^ = 87%; *p* < 0.01; Supplementary Figure S9).

### Secondary outcome: functional prognosis

**Figure 3:**
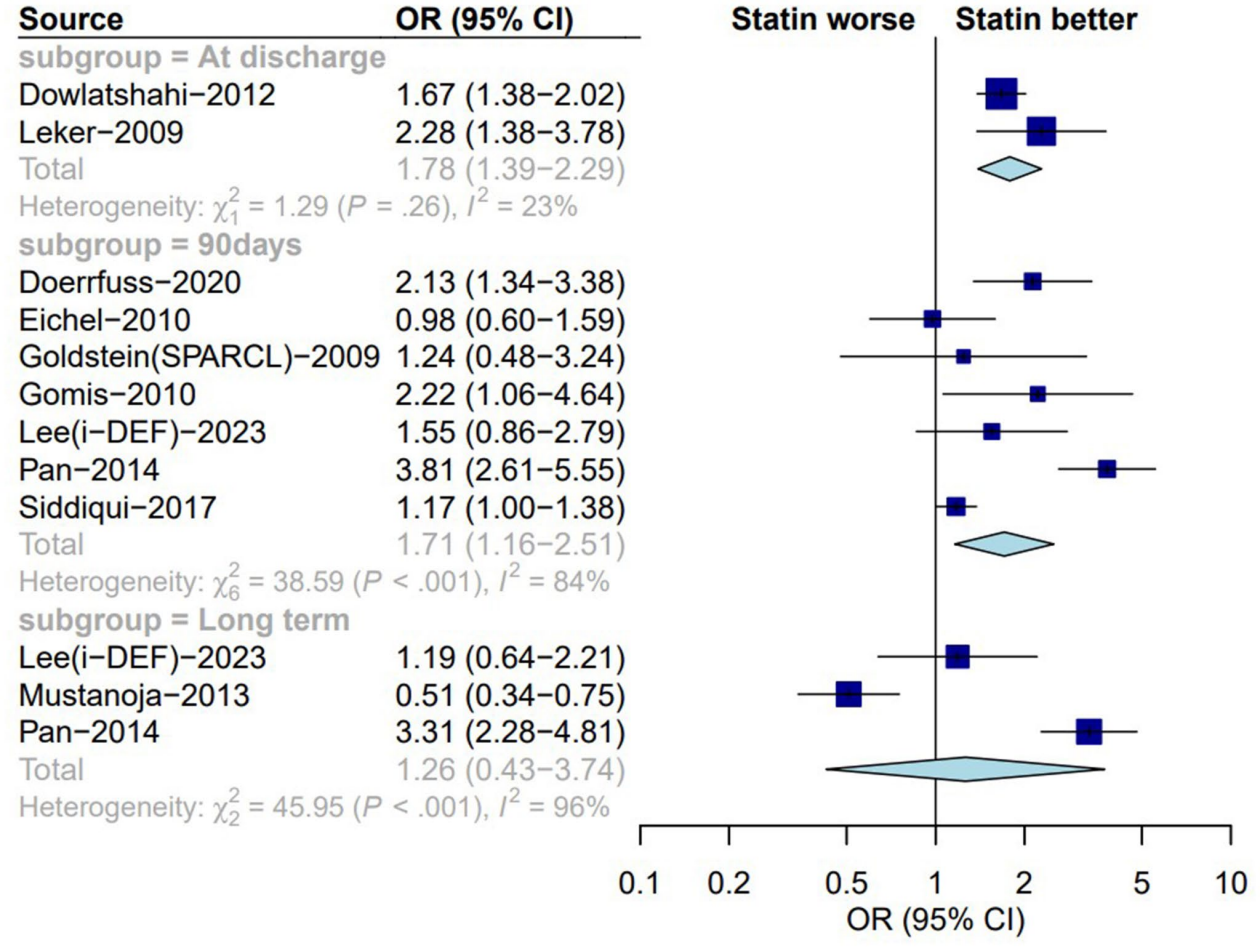
Forest plot of functional prognosis between statin group and non-statin group. OR, odds ratio; CI, confidence interval.

Ten of the studies (5, 14, 18, 22, 25, 29, 31, 34, 35, 37) selected for analysis included secondary outcome indicators, including a good functional prognosis. Our analysis revealed that the administration of statins at different timepoints improved the prognosis of patients with ICH. There was no significant difference between the statin and no-statin groups in terms of achieving a better functional prognosis at discharge (OR: 1.78; 95% CI: 1.39–2.29; *I*^2^ = 23%; *p* < 0.01; Figure 3). Ninety days of statin therapy after ICH was associated with better functional outcomes than no statin therapy (OR: 1.71; 95% CI: 1.16–2.51; *I*^2^ = 84%; *p* = 0.01; Table 1). Long-term analysis (6 months to 1 year) showed that those who received statin therapy achieved better functional outcomes than those who did not receive statin therapy (OR: 1.26; 95% CI: 0.43–3.74; *I*^2^ = 96%; *p* = 0.71; Table 1).

**Table 1:** Outcomes of meta-analysis.

Outcomes	OR	95% CI	*p*-value	Heterogeneity test	
				*I*^2^%	PH
**Mortality**					
Total mortality	0.61	0.51–0.73	*p* < 0.01	87	*p* < 0.001
Subgroup					
In-hospital	0.63	0.46–0.88	*p* < 0.01	74	*p* < 0.001
30 days	0.52	0.29–0.92	*p* = 0.03	93	*p* < 0.001
90 days	0.62	0.47–0.82	*p* < 0.01	70	*p* < 0.001
Long term	0.54	0.33–0.91	*p* = 0.02	91	*p* < 0.001
Design					
PCS	0.56	0.40–0.77	*p* < 0.01	89	*p* < 0.001
RCS	0.64	0.51–0.81	*p* < 0.01	87	*p* < 0.001
PSM	0.59	0.48–0.74	*p* < 0.01	90	*p* < 0.001
**Functional prognosis**					
At discharge	1.78	1.39–2.29	*p* < 0.01	23	*p* = 0.26
90 days	1.71	1.16–2.51	*p* = 0.01	84	*p* < 0.001
Long term	1.26	0.43–3.74	*p* = 0.71	96	*p* < 0.001

PCS, prospective cohort study; RCS, retrospective cohort study; PSM, propensity score matching; OR, odds ratio; CI, confidence interval.

### Sensitivity analyses

In order to verify the stability of the meta-analysis results, we reanalyzed the results using the “leave one out” method of R software. We conducted sensitivity analyses for the total mortality associated with the use of statins by excluding one study from our analysis at a time followed by re-evaluation. One study (13) excluded from our analysis that had the greatest impact on the OR for statin on total mortality. Analysis of the remaining studies showed that the exclusion of this particular study did not affect the outcome of statin use on total mortality (OR: 0.63; 95% CI: 0.52–0.75; *I*^2^ = 84%; *p* < 0·01; Supplementary Figure S19). In addition, the study of other groups did not have a significant impact on the overall results (Supplementary Figures S10–S27).

### Heterogeneity analysis

To further explore the potential sources of heterogeneity, we conducted subgroup analyses on the effect of statin use on overall mortality in patients with intracerebral hemorrhage. The subgroups included: age (>65 years vs. ≤65 years), study center (single-center vs. multi-center), study design (prospective cohort study [PCS] vs. retrospective cohort study [RCS]), propensity score matching (PSM: Yes vs. No), and publication year (before 2015 vs. after 2015). However, the results demonstrated that the heterogeneity remained substantial across all subgroup analyses, indicating that these factors did not significantly contribute to the observed heterogeneity (Supplementary Figures S28–S32).

## Discussion

Compared to previous publications, this meta-analysis has a number of advantages, including a sufficient sample size, the analysis of subgroups by propensity score matching (PSM), and a more efficient means of controlling for confounding factors. Over the past decade, a large amount of data have been collated by multiple centers; this has allowed prospective cohort studies to include more comprehensive designs and allowed for the publication of many randomized controlled trials (RCTs). Since 2019, nine high-quality studies have been published, accounting for one-third of the total number of articles included in the present analysis. Furthermore, some of the existing meta-analyses relating to the impact of statins on the prognosis of ICH included subgroup analyzes at different timepoints after the onset of ICH. But these existing studies relating to the prognosis of patients with ICH, including total mortality and functional prognosis, did not consider design and PSM as indicators for subgroup analysis. Therefore, in the current meta-analysis, we aimed to investigate whether statins can exert adverse effects on the prognosis of patients with ICH by performing objective and systematic analyses.

In the present study, we comprehensively searched several online databases and identified relevant literature by formulating strict inclusion and exclusion criteria. Finally, we screened 30 high-quality publications involving 42,298 patients, including 16,482 cases in the statin group and 25,816 cases in the non-statin group. We extracted key data from the literature and performed statistical analysis with Revman version 5.3 software and R version 4.3.2.

The results of our meta-analysis showed that statin treatment significantly reduced the total mortality of patients after ICH (OR: 0.61; 95% confidence interval: 0.51–0.73; *p* < 0.01); these findings are consistent those reported by previous meta-analyses (8, 10). In addition, we performed subgroup analysis to reduce the influence of confounding factors. Our outcomes showed that statins reduced the mortality of patients with ICH after different time periods (after 30 days, 90 days and long-term hospitalization), thus indicating that statins play a protective role from the onset of ICH to a long-term prognosis. In order to exclude the influence of confounding factors, our subgroup analysis included a PSM group (OR: 0.59; 95% CI: 0.48–0.74; *p* < 0.01). Our analysis showed that statins still played a protective role after the exclusion of confounding factors, such as age, hypertension, and diabetes. In order to avoid the impact of different research designs on our results, we performed subgroup analysis according to different trial designs. Analysis showed that PCS and RCS both reduced mortality after ICH.

In this study, we selected a good functional prognosis at each stage after ICH as the evaluation index, and good functional recovery was defined as the patient being able to walk independently (an mRS of 0–3 points). Our meta-analysis results showed that statins can reduce the degree of disability at different periods (at discharge and 90 days). However, there was low certainty evidence indicating that the use of statins could achieve better a prognosis over the long-term.

Historically, researchers have considered that the adverse effects of statins on patients with ICH may arise from two main aspects. Firstly, statins reduce the plasma levels of cholesterol and increase the permeability of the blood–brain barrier. Epidemiological studies have reported that hypocholesterolemia can increase the incidence and mortality of hemorrhagic stroke and that adequate cholesterol levels are necessary to maintain the integrity of the cerebral vessels (40). A previous cohort study by Xie et al. (41) confirmed that hypolipidemia (low-density lipoprotein cholesterol (LDL-C) < 70 mg/dL) represents an independent high-risk factor for severe hemorrhagic stroke (42). Secondly, statins inhibit the coagulation cascade by exerting anti-platelet and fibrinolytic effects and by influencing coagulation factors, thus causing expansion of a cerebral hematoma. However, the pleiotropic effects of statins (43), such as anti-inflammation, increased cerebral blood flow, resistance against oxidative stress, reduced cerebral edema, and other protective effects on the nervous system, cannot be ignored. Our meta-analysis showed that statins improve the prognosis of patients with ICH at different time points. Several factors may explain why statins can exert protective effects on the nervous system at different times after ICH. For example, during the acute phase of cerebral hemorrhage (in-hospital), statins can reduce perihematomal edema (PHE), resist oxidative stress and inflammation, and reduce extensive neuronal damage during the recovery period (30–90 days after onset). During this recovery period, statins improve cerebral blood flow, increase cerebral perfusion, and correct the central nervous system. In the long-term (6 months to 1 year) after the onset of ICH, statins promote vascular endothelial growth, nerve regeneration, stabilize the blood–brain barrier, and prevent the recurrence of stroke.

The Heart Protection Study (HPS) (44) and SPARCL studies (4) confirmed that statins can significantly reduce the incidence of ischemic cardiovascular and cerebrovascular diseases. The main findings of the SPACL study showed that statins significantly reduced the incidence of ischemic strokes and transient ischemic attacks and conducted secondary analyzes based on first outcome hemorrhagic events. The initial trial reported that statins increased the risk of cerebral hemorrhage, although the SPARCL secondary analysis (5) (*n* = 43; including 29 patients taking atorvastatin and 14 patients on placebo) was obviously limited by a small sample size and low statistical power. Furthermore, the SPARCL trial used a high dose of atorvastatin dose (80 mg/d); high doses of statins can significant reductions in blood lipids. However, an excessive reduction of blood lipid levels can increase the risk of cerebral hemorrhage and may also cause muscle and liver damage. For secondary prevention, the most commonly used dose of atorvastatin to control blood lipid levels in stroke patients is small to medium (20–40 mg/d). With the exception of the SPACRCL study, no previous trial has produced convincing evidence to confirm that statins worsen the prognosis of patients with ICH. Although statins have a double-edged sword effect of anti-atherosclerosis and increased bleeding risk, more attention should be paid to the overall condition of each patient. For example, if an ICH patient also suffers from hyperlipidemia, coronary heart disease, ischemic stroke, etc., it is recommended to actively give statins for treatment.

### Strengths and limitations

Our study has several strengths: it includes a large sample size with 30 studies, one-third of which were published in the past 5 years. Additionally, it features more refined subgroup analyses and a comprehensive sensitivity analysis. Although our present analysis included 14 new studies and conducted a more comprehensive subgroup analysis when compared with the previous meta-analysis reported by Jung et al. (8), we were still unable to acquire sufficient data relating to the types and dosages of statins. Furthermore, we were unable to analyze additional factors such as the dissolution characteristics and dosage of statins with regards to their impact on the prognosis of patients with ICH. Not all subjects adhered to their randomized treatment and we were unable to consider socioeconomic status, immediate poststroke complications, detailed cognitive function, family support, insurance status, and length of stay in hospital. Rehabilitation data cannot control the influence of different factors on experimental design. In general, there is currently a lack of large-scale RCTs relating to the clinical application of statins for patients with cerebral hemorrhage to fully identify the impact of statins on the prognosis of cerebral hemorrhage. The SATURN trial (NCT03936361) is a 6-year RCT study involving 1,456 patients with intracerebral hemorrhage. Data derived from this trial will further reveal the impact of continuous or discontinuous statin administration on the prognosis of ICH.

## Conclusion

Our meta-analysis suggests that statin use may be associated with improved mortality and functional outcomes in patients with intracerebral hemorrhage (ICH). However, due to the observational nature of the included studies and the lack of randomized controlled trial (RCT) data, the evidence remains insufficient to establish a definitive causal relationship. Further studies, including RCTs, are needed to better understand the effects of different statin types, dosages, and durations on ICH outcomes. Additionally, more comprehensive data, including age and sex stratification, are required to refine clinical recommendations and fully assess the potential benefits of statins in this patient population.

## Data Availability

The original contributions presented in the study are included in the article/Supplementary material, further inquiries can be directed to the corresponding authors.
